# Increased mitochondrial fission is critical for hypoxia-induced pancreatic beta cell death

**DOI:** 10.1371/journal.pone.0197266

**Published:** 2018-05-16

**Authors:** Da Zhang, Yanfang Liu, Yao Tang, Xiaofeng Wang, Zhichao Li, Rui Li, Zhenyu Ti, Weidong Gao, Jigang Bai, Yi Lv

**Affiliations:** 1 Department of Hepatobiliary Surgery, Institute of Advanced Surgical Technology and Engineering, Shaanxi Center for Regenerative Medicine and Surgical Engineering, The First Affiliated Hospital of Xi’an Jiaotong University, Xi’an, China; 2 Department of General Surgery, Xi'an No.3 Hospital, Xi’an, China; 3 Department of Ophthalmology, Xi'an Children’s Hospital,Xi’an, China; Texas Technical University Health Sciences Center, UNITED STATES

## Abstract

Hypoxia-mediated pancreatic beta cell death is one of the main causes of pancreatic beta celldeath, which leads to the loss of functional pancreatic beta cell mass and type 1 diabetes andtype 2 diabetes.However, the molecular mechanisms that control life and death of pancreatic beta cells remain poorly understood. Here we showed that mitochondrial fission was strongly induced in pancreatic beta cellsmainly due to an elevation of DRP1^S616^ phosphorylation through HIF-1αactivation and subsequent DRP1 mitochondrial translocation. Hypoxia-induced pancreatic beta cell death can be reversed by the inhibition of mitochondrial fission viaDRP1 knockdown. We further demonstrated that hypoxia-induced mitochondrial fission untightened the cristae formation, which subsequently triggers mitochondrial cytochrome c release and consequent caspase activation. Moreover, treatment with mitochondrial division inhibitor-1 (Mdivi-1), a specific inhibitor of DRP1-mediated mitochondrial fission, significantly suppressedbeta cell death in vitro, indicating a promising therapeutic strategy for treatment of diabetes.Taken together, our results reveal a crucial role for the DRP1-mediated mitochondrial fission in hypoxia-induced beta cell death, which provides a strong evidence for thisprocess as drug target indiabetestreatment.

## Introduction

A fundamental challenge in treating diabetes is theidentification of the molecular basesthat cause beta cell failure in response to environmental stress factors, including hypoxia. More and more studies support that pancreatic betacells are heavily dependenton mitochondrial respiration and commonly sensitive to hypoxic stress due to their high consumption of oxygen during insulin secretion[1, 2].Hypoxia-mediated cell death is still one of the main problems that must be solved for transplantation to be regarded as a reliable therapy [3].However, the molecular mechanisms behind this are poorly understood.

Mitochondria are multifunctional and highly dynamic organelles, which are regulated by constant fusion and fission events[4]. Balanced fusion and fission is critical for appropriate numbers, morphology and activity of mitochondrial to satisfy the variable need of cells and adapt tothe cellular environment[5].To date, severalcore components of fusion and fission machineryhave now been identified, includingmitofusins (MFN1 and MFN2) and optic atrophy 1 (OPA1) for mitochondrial fusion and dynamin-related protein 1 (DRP1), mitochondrial fission 1 protein (Fis1) and mitochondrial fission factor (MFF)for mitochondrial fission[6].Moreover, recent studies have indicated that mitochondrial fission and fusion play a role in the regulation of cell apoptosis, showing thatincreased mitochondrial fusion suppresses apoptosis, whereas elevation in fission favorsapoptosis[7–9]. However, we still do not know the role of mitochondrial fusion and fission in hypoxia-induced pancreatic betacell death.

It is wellknown that cytochrome c release from mitochondria to cytosolic is a critical step to cell death, whichsubsequentlyresulted in caspase activation and apoptotic cell death[10, 11]. Cristae are folded structures that greatly increase the total surface area of the inner membrane of mitochondria, providing more space for the series of compounds such as respiratory chain includingcytochrome c[12, 13]. A previous study showed that OPA1-mediated mitochondrial fusioncontributesto cristae reformation and cytochrome c release inhibition[14], implicating that mitochondrial dynamic-regulated cristae remodeling plays a critical role in cell apoptosis regulation.

Here we investigated the changes of mitochondrial morphology in pancreatic beta cells and theirfunctional roles in the regulation of celldeath and survival during hypoxia situations(1% O_2_). Moreover, the underlying mechanisms and therapeutic applicationwas systematically explored.

## Materials and methods

### Cell culture

Rat insulinoma cell line INS-1E, a gift from Dr. P. Maechler (University of Geneva, Switzerland), was cultured in RPMI-1640 media supplemented with 10% fetal bovine serum (Hyclone), 50 μmol/l β-mercaptoethanol, 1mmol/l sodium pyruvate, 50 U/ml penicillin and 50 μg/ml streptomycin. For DRP1 silencing, INS-1E cells were transfectedwith siRNA against DRP1(5'-CUACUUCCUGAAAACAAC-3') or scrambled siRNA (5'-AATTCTCCGAACGTGTCACGT-3') for 48 h using lipofectamine 2000 (Invitrogen, Carlsbad, CA, USA) according to the manufacturer’sinstructions.

### Antibodies and reagents

The following primary antibodies were used in this study: anti-MFN1 (cat. #ab104585, Abcam), anti-MFN2 (cat. #ab101055, Abcam), anti-OPA1 (cat. #ab90857, Abcam), anti-DRP1 (cat. #ab56788, Abcam), anti-Fis1 (cat. #ab156865, Abcam), anti-MFF (cat. #ab125079, Abcam), anti-DRP1(S616) (cat. #4494, Cell Signaling), anti-DRP1(S637) (cat. #4867, Cell Signaling), anti-HIF-1α (cat. 610958, BD Biosciences), anti-CDAC (cat. #ab14734, Abcam), anti-β-actin (cat. TDY041, Beijing TDY BIOTEC) and horseradish peroxidase-conjugated anti-rabbit (cat. S004, Beijing TDY BIOTEC) and anti-mouse secondary antibody (cat. S001, Beijing TDY BIOTEC). The DRP1 inhibitor Mdivi-1was purchased from Sigma-Aldrich (cat. M0199).

### Transmission electron microscopy (TEM) for mitochondrialmorphology analysis

For conventional TEManalysis, pancreatic beta cellswere firstly fixed by glutaraldehyde. Then thecells were OsO4 post-fixed, alcohol dehydrated, andembedded in araldite. After staining with uranylacetate and lead citrate, sections were analyzed by using a Tecnai G2 electronmicroscope (FEI, cHillsboro, Oregon). In addition, imageJ software (NIH, Bethesda, MD) was used for mitochondrialength and cristaewidth analysis.

### Mitochondrial morphology analysis by confocal microscopy

For fluorescence analysis of mitochondrial morphology by confocal microscopy, The fluorescent dye MitoTracker green FM (Molecular Probes, M7514) was used to monitor mitochondrial morphology in pancreatic beta INS-1E cells according to the manufacturer’s instructions. Then cells were viewed and photographed with an Olympus FV 1000 laser-scanning confocal microscope (Olympus Corporation, Tokyo, Japan). The length of mitochondria was measured using the ImageJ software. A cell with fewer than 25% of the mitochondria visible in the cell had a length five times its width was judged to have fragmented mitochondria. A cell with greater than 75% of the mitochondria had a length five times its width highly was judged to have tubulated mitochondria interconnected if greater than 75% of the mitochondria had a length five times its width. Greater than 50 cells per group were blindly analyzed by three people.

### Mitochondrial isolation

Acell mitochondria isolation kit (Beyotime, Nantong, China) was used for isolation of mitochondria from pancreatic betaINS-1E cellsaccording to the manufacturer’s instructions. Firstly, cells were washedtwice with ice-cold PBS and resuspended in mitochondrial isolation buffer on ice for 20 min. Then, cells were centrifuged at 600 g for 10 min in 4°C before fully homogenized. The supernatant wascentrifuged again in another tube at 11,000 g for 10 min in 4°C. At last, pellet was resuspended in mitochondria storage buffer.

### Real-time PCR and western blot analysis

Total RNA was extracted with Trizol reagent (Invitrogen, Carlsbad, CA) and then reverse transcribed into cDNAs by using PrimeScript® RT Reagent Kit (Takara). The amplifications were carried out using the SYBR Green PCR Master Mix (Applied Biosystems). The primers used in this study were: MFN1 sense 5'-TGGCTAAGAAGGCGATTACTGC-3', and antisense, 5'-TCTCCGAGATAGCACCTCACC-3'; MFN2 sense 5'-CTCTCGATGCAACT-CTATCGTC-3', and antisense, 5'-TCCTGTACGTGTCTTCAAGGAA-3'; OPA1 sense 5'-TGTGAGGTCTGCCAGTCTTTA-3', and antisense, 5'-TGTCCTTAATTGGGGTCGTTG-3'; DRP1 sense 5'-GGAGACTCATCTTTGGTGAAGAG-3', and antisense, 5'-AAGGAGCCAGTCAAATTATTGC-3'; Fis1 sense 5'-GTCCAAGAGCACGCAGTTTG-3', and antisense, 5'-ATGCCTTTACGGATGTCATCATT-3'; MFF sense 5'-ACTGAAGGCATTAGTCAGCGA—3', and antisense, 5'-TCCTGCTACAACAATCCTCTCC-3'; GAPDH sense 5'-CGGAGTCAACGGATTTGGTCG-3', and antisense 5'-AGCCTTCTCCATGGTGGTGAAGAC-3', was used as control. The relative quantification in gene expression was determined by using the 2^-ΔΔC^t method.

For western blot analysis, proteins were separated with 8% or 10% SDS-polyacrylamide gel electrophoresis and then transferred to a PVDF membrane. After blocked with 5% non-fat milk, the membranes were incubated with primary antibodies overnight at 4°C and HRP-conjugated secondary antibody for 2 h at 28°C. Blots were washed and detected by western maximumsignal sensitivity kit (Pierce).

### MTS assay

MTS assay was used for cell viability determination. Briefly, cells were collected and wash by PBS, and then incubated in 20ml of MTS (0.2%)-PMS (0.092%) solution for 2 h. Absorbance was detected at 490 nm. Eachsample was analyzed in triplicate.

### Apoptosis assays

Abcam's Annexin V-FITC Apoptosis Staining/Detection Kit was used for cell apoptosis analysis by flow cytometry. Briefly, pancreatic beta INS-1E cells were collected and resuspended by500 ml binding buffer with 5 ml ANXA5-FITC and 5 ml PI. Then, cells were incubated for 15 min at room temperature in thedark and analyzed with a flow cytometer(Beckman, Fullerton, CA). TUNELassay (Roche, 11684795910) was also used for analysis of apoptosis inpancreatic beta cells following the manufacturer's instructions. Images were obtained by a fluorescence microscope.

### Statistical analysis

The data represent mean ± SD.All experimentswererepeated 3 times. SPSS16.0softwarewasusedforallstatisticalanalyses. Comparisons between two groups were performed with an unpaired two-tailed Student’s t test and multiple group comparisons were performed by unpaired one-way ANOVA followed by post hoc Turkey’s test. Differences of a *P*-value<0.05 were considered as statistically significant.

## Results

### Hypoxiainduces mitochondrial fragmentation in pancreatic beta cells

**Fig 1:**
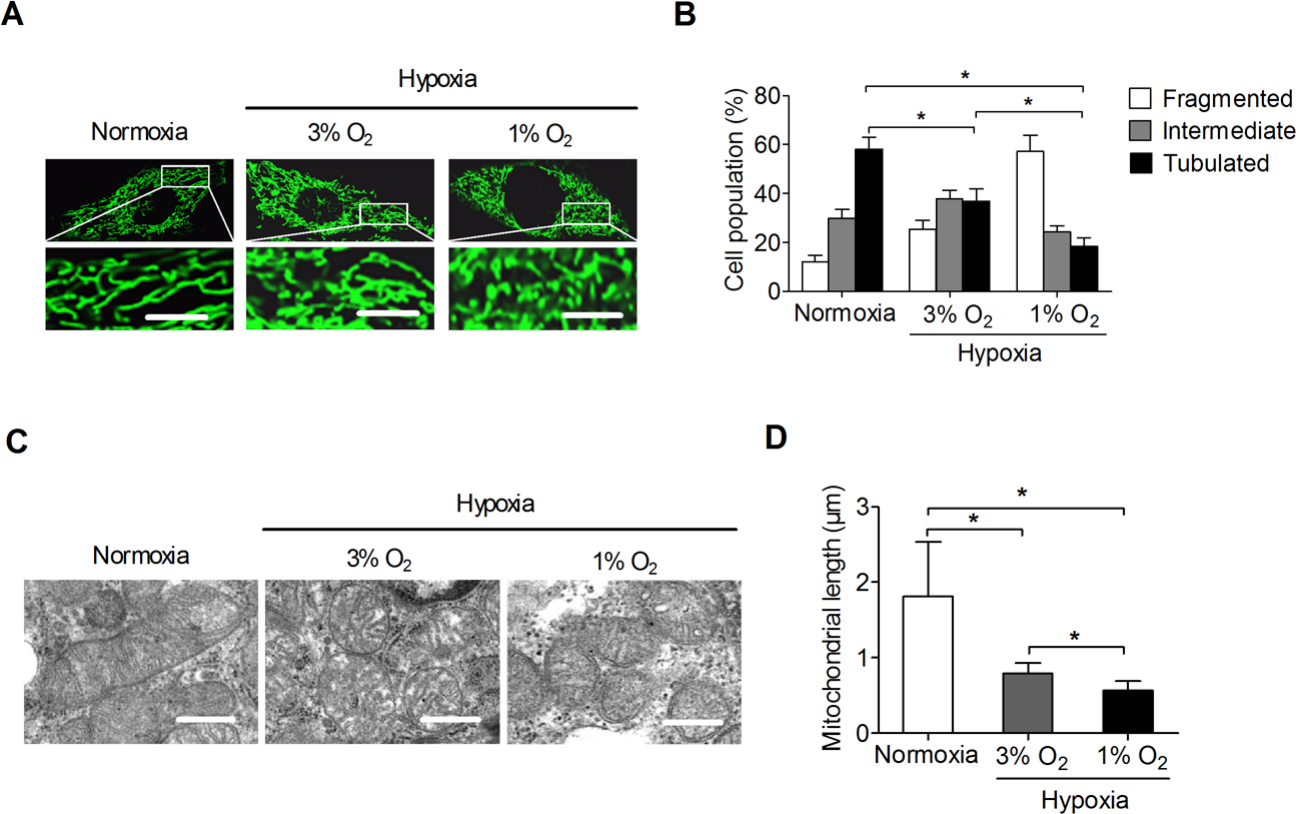
Hypoxia induces mitochondrial fragmentation in pancreatic beta INS-1E cells. **A.** Representative confocal microscope images of the mitochondrial in pancreatic beta INS-1E cellsafter treatment with hypoxia at different oxygen levels (3% or 1% O_2_ for 24 h) as indicated. Scale bars, 10 μm.**B.**The proportion of pancreatic beta INS-1E cellswith tubulated, intermediate, and fragmented mitochondria was quantified. **C.** Representative transmission electron microscopy (TEM) images of the mitochondrialin INS-1Ecells treated as indicated. Scale bars, 0.5 μm. **D.**Image J was used for mitochondrial length quantification in INS-1Ecells treated as indicated.All experiments were repeated three times.*P< 0.05.

To investigate the impact of hypoxia on the mitochondrial dynamics in pancreatic beta cells, the alterations of mitochondrial morphologywere examined by staining with mitochondrial dye,MitoTracker Green.Fluorescence microscopy analysis showed that hypoxia(1% or 3% O_2_) treatment resulted in a dramatic increase of mitochondrial fragmentationin pancreatic beta INS-1Ecells, with a higher increase at 1% O_2_ than at 3% O_2_of mitochondrial, indicating adose-dependent manner (Fig 1A and 1B).Transmission electronmicroscopy (TEM)further revealed a significantly lower average mitochondrial lengthin pancreatic beta INS-1Ecells with hypoxia(1% or 3% O_2_) treatment compared with those under normoxia (Fig 1C and 1D).

### HIF-1α activation and subsequently DRP1(S616) phosphorylationis involved in hypoxia-induced mitochondrial fragmentation in pancreatic beta cells

**Fig 2:**
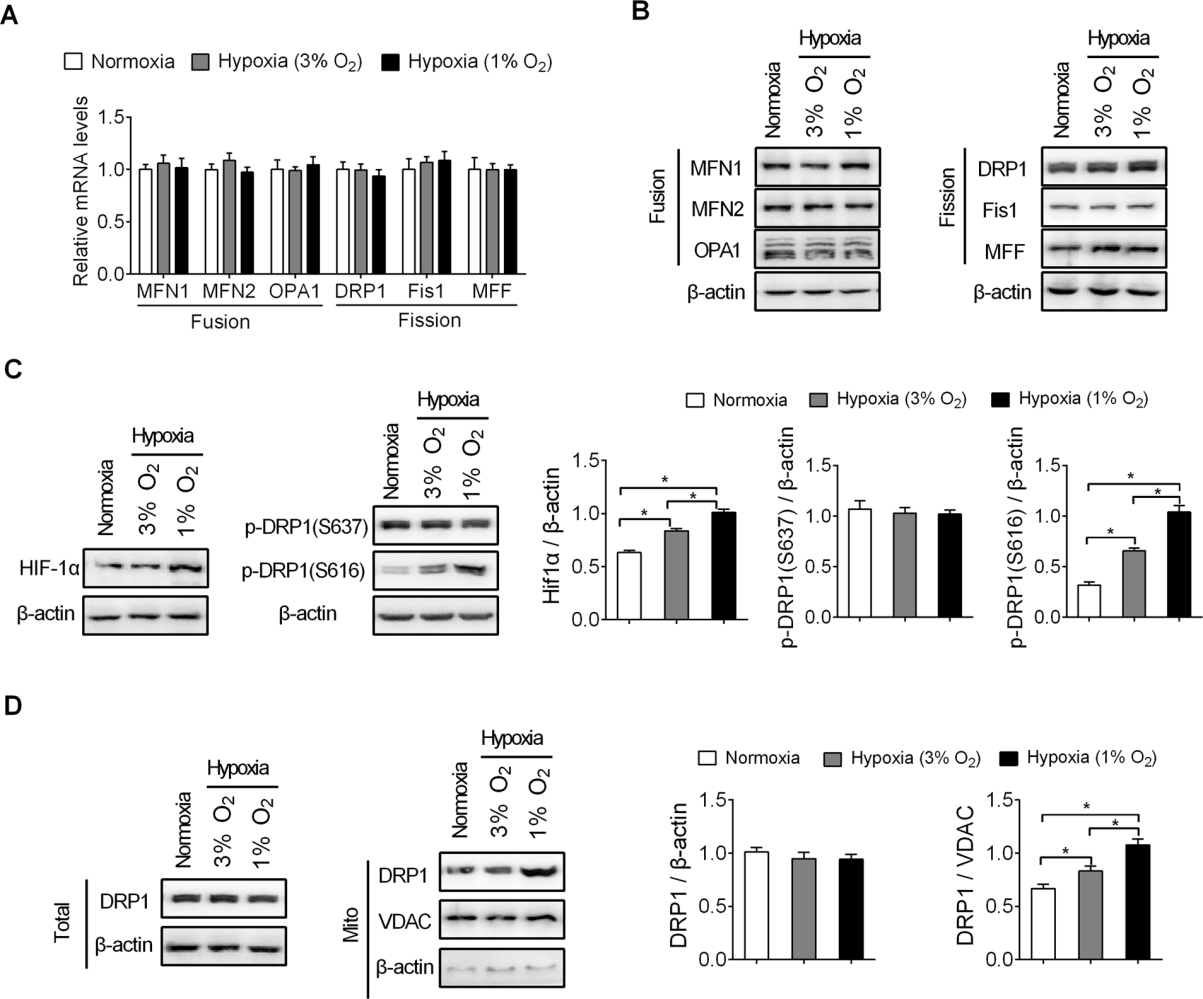
HIF-1α activation and subsequently DRP1(S616) phosphorylationis involved in hypoxia-induced mitochondrial fragmentation in pancreatic beta cells. **A-B.** Expression levels of MFN1, MFN2, OPA1, DRP1, Fis1, and MFF were detected by RT–PCR (A) and western blot (B)analysis in pancreatic beta INS-1E cellswith treatment as indicated. **(C)**Top: Levels of HIF-1α and phosphorylated DRP1 (S637)or(S616)were detected by western blot analysis in pancreatic beta INS-1E cellswith treatment as indicated.Bottom: Western blot scanning densitometry for three independent experiments.Blots were probed for β-actin to ensure equal proteinloading. *P<0.05.**(D)**Top: Levels of DRP1 in total lysates and mitochondrial fraction were examined by western blot analysis in pancreatic beta INS-1E cellswith treatment as indicated. VDACserves as loading controls. Mito: mitochondria.Bottom: Western blot scanning densitometry for three independent experiments.Blots were probed for β-actinor VDAC respectively to ensure equal proteinloading. *P<0.05.All experiments were repeated three times.

To explore the underlying mechanism of hypoxia-induced mitochondrial fragmentation in pancreatic beta cells, we analyzed the expression levels of core mitochondrial dynamic mediators, including MFN1, MFN2 and OPA1 for mitochondrial fusion and DRP1, Fis1 and MFF for mitochondrial fissionunder hypoxia conditions by RT-PCR and western blot analysis. Surprisingly,no significant expression changes of MFN1, MFN2, OPA1, DRP1, Fis1 andMFF wereobserved at either the mRNA or protein level in pancreatic beta INS-1E cells with hypoxia treatment as compared to those under normoxia (Fig 2A and 2B).It is well known that hypoxia-inducible transcription factor (HIF-1α) is a major regulator of cellular adaptation to low oxygen stress. A previous study has shown HIF-1αactivation can promote mitochondrial fission by phosphorylation of DRP1 at Serine-616[15], a critical post-translational modification of DRP1 for its translocation from cytoplasm to mitochondrial. Therefore, we hypothesized that hypoxia-mediated HIF-1αactivation and subsequently DRP1(S616) phosphorylationmay be involved in the regulation of mitochondrial fragmentation by hypoxia in pancreatic beta cells. As shown in Fig 2C, a dramatic increase of both HIF-1α and DRP1(S616) phosphorylationwere observed inpancreatic beta INS-1E cells upon hypoxia treatment. While no significant changein thephosphorylation of DRP1at Serine-637, an inhibitory modification for fission activity of DRP1,was detectedupon treatment with hypoxia. Moreover,the level of DRP1 in the mitochondrial fractions was significantly increased upon hypoxia treatment, further confirming the importance of DRP1(S616) phosphorylation in hypoxia-induced mitochondrial fragmentation of pancreatic beta cells(Fig 2D).

### Mitochondrial fragmentation is involved inpancreatic beta cell death in hypoxia conditions

**Fig 3:**
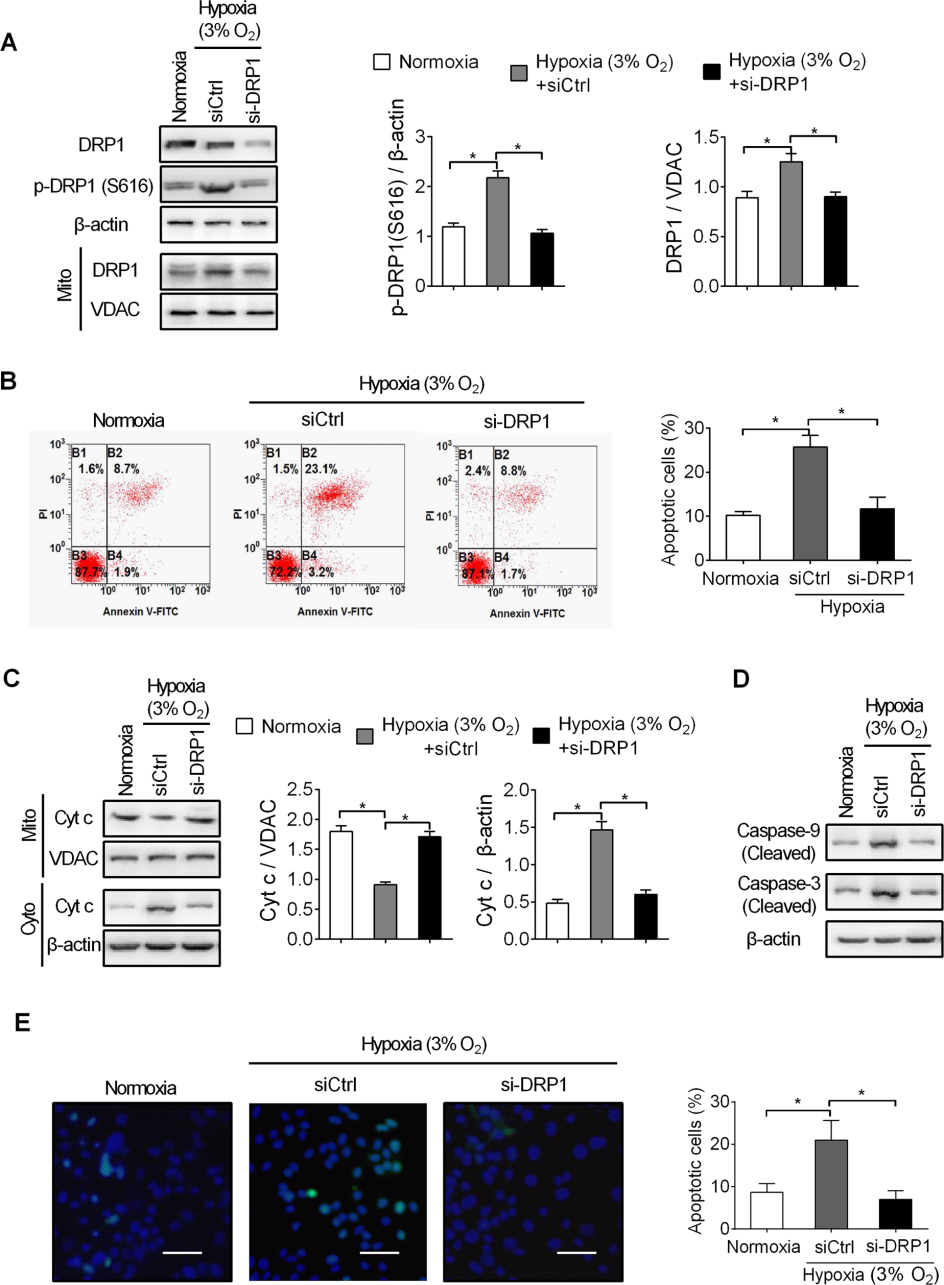
Mitochondrial fragmentation is involved inpancreatic beta cell death in hypoxia conditions. **A.**Detection of DRP1 (S616) and mitochondrial-fractional DRP1by western blot analysis in pancreatic beta INS-1E cellswith treatment as indicated. **B.**Detection of apoptosis by flow cytometry in pancreatic beta INS-1E cellswith treatment as indicated. **C.**Western blot analysis for levels of cytochrome c inpancreatic beta INS-1E cellswith treatment as indicated. β-actin and VDAC were used as loading controls for cytoplasm and mitochondria, respectively. Cyto, cytoplasm; Mito, mitochondrial. **D.**Western blot analysis for levels ofcleaved Caspase-9 and Caspase-3 inpancreatic beta INS-1E cellswith treatment as indicated. **E.**TUNEL staining in pancreatic beta INS-1E cells from type 2 diabetes animal models. Blue: Hoechst 33342; Green: TUNEL positive nucleus. All experiments were repeated three times.*P< 0.05.

To determine the biological effectsof hypoxia-induced mitochondrial fragmentation on pancreatic betacells, cell apoptosis was assessed in pancreatic beta INS-1E cellswith inhibited mitochondrial fission by DRP1 knockdown under hypoxia. As shown in Fig 3A, hypoxiapromoted phosphorylation of DRP1(S616) can be attenuated by DRP1 knockdown. In addition, hypoxia significantly inducedcell apoptosis of pancreatic beta INS-1E cells when compared with those under normoxia. Whereas inhibition of the mitochondrial fission by knockdown of DRP1 significantly restoredhypoxia-induced apoptosis (Fig 3B), indicating that DRP1 (S616)phosphorylation-mediated mitochondrial fragmentation is an upstream factor forpancreatic beta cell death during hypoxia conditions.Furthermore, release of cytochrome c from mitochondria into cytosol, and cleavage ofcaspase 3 and caspase 9were significantly induced by hypoxia treatment, whereas all of themwere remarkably inhibitedwhen DRP1 was knocked-down in pancreatic beta INS-1E cells (Fig 3C and 3D). Moreover, a marked increase ofpositive TUNEL (transferase–mediateddUTP nick-end labeling) stainingwas observed in pancreatic beta cells under hypoxia conditions. In contrast,the pro-apoptotic effects of hypoxia treatment were significantly reversed by inhibition of mitochondrial fission by DRP1 knockdown (Fig 3E). Collectively, these results indicate that mitochondrial fragmentation playsan essential role in promoting hypoxia-induced pancreatic beta cell death, and inhibition of mitochondrial fission by DRP1 knockdown could protect pancreatic beta cell against hypoxia-induced cell death.

### Hypoxia-induced mitochondrial fragmentationfacilitatescristae remodeling in pancreatic beta cells

**Fig 4:**
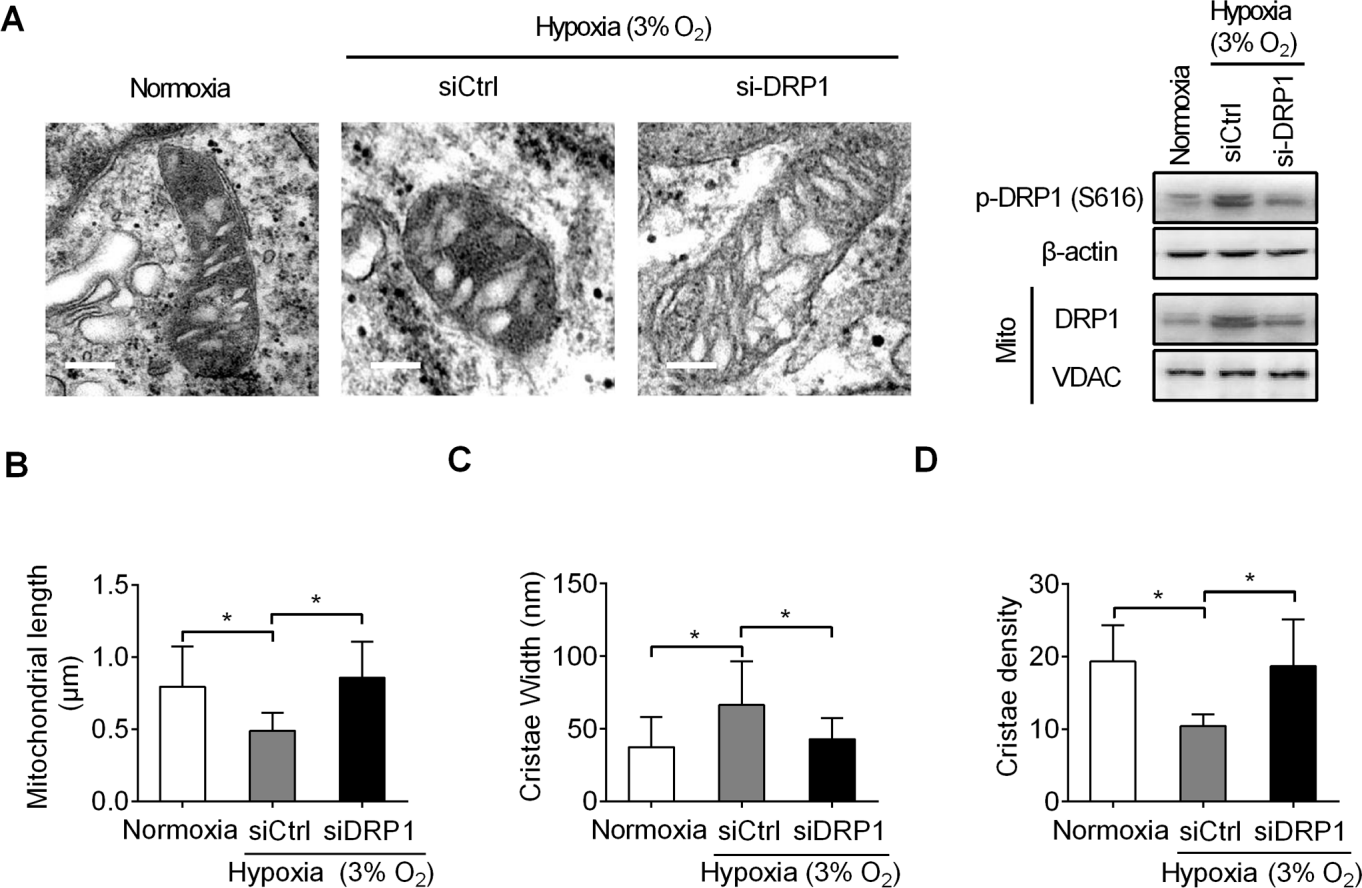
Hypoxia-induced mitochondrial fragmentation facilitates cristae remodeling in pancreatic beta cells. **A.**Representative transmission electron microscopy (TEM) images of mitochondria in pancreatic beta INS-1E cells treatedas indicated.Scale bars, 0.1 μm.**B-D.** Mitochondrial length (B), cristae width (C) and cristae density(number/mitochondrial length)(D)were quantitatively analyzedin pancreatic beta INS-1E cells treatedas indicated. Over twenty mitochondria were randomly selected for quantitative analysis in each treatment group.All experiments were repeated three times.*P< 0.05.

A previous study has shown that OPA1-mediated mitochondrial fusion promotes cristaereformation and inhibits cytochrome c release[14], implying a critical role for cristae remodeling in cell apoptosis regulation. We thus examined the effect of hypoxia-induced mitochondrial fragmentation on the structure remodeling of cristae by using transmission electronmicroscopy (TEM). Representative transmission electron microscopy (TEM) images in Fig 4A showed that hypoxia significantly induced mitochondrial fragmentation in pancreatic beta cells,while DRP1 knockdown clearly reversed hypoxia-induced mitochondrial fragmentation(Fig 4B). Moreover, pancreatic beta cells with hypoxia treatment showed a significant increase of cristae width(Fig 4C) and reduction of cristaedensity, all of which could be reversed by DRP1 knockdown(Fig 4D). These results collectively indicate that mitochondrial fragmentation-mediatedcristaeremodelingshould be, at least in part, involved inhypoxia-induced pancreatic beta cell death.

### Mdivi-1 exhibits a therapeutic effecton hypoxia-induced cell death of pancreatic beta cells

**Fig 5:**
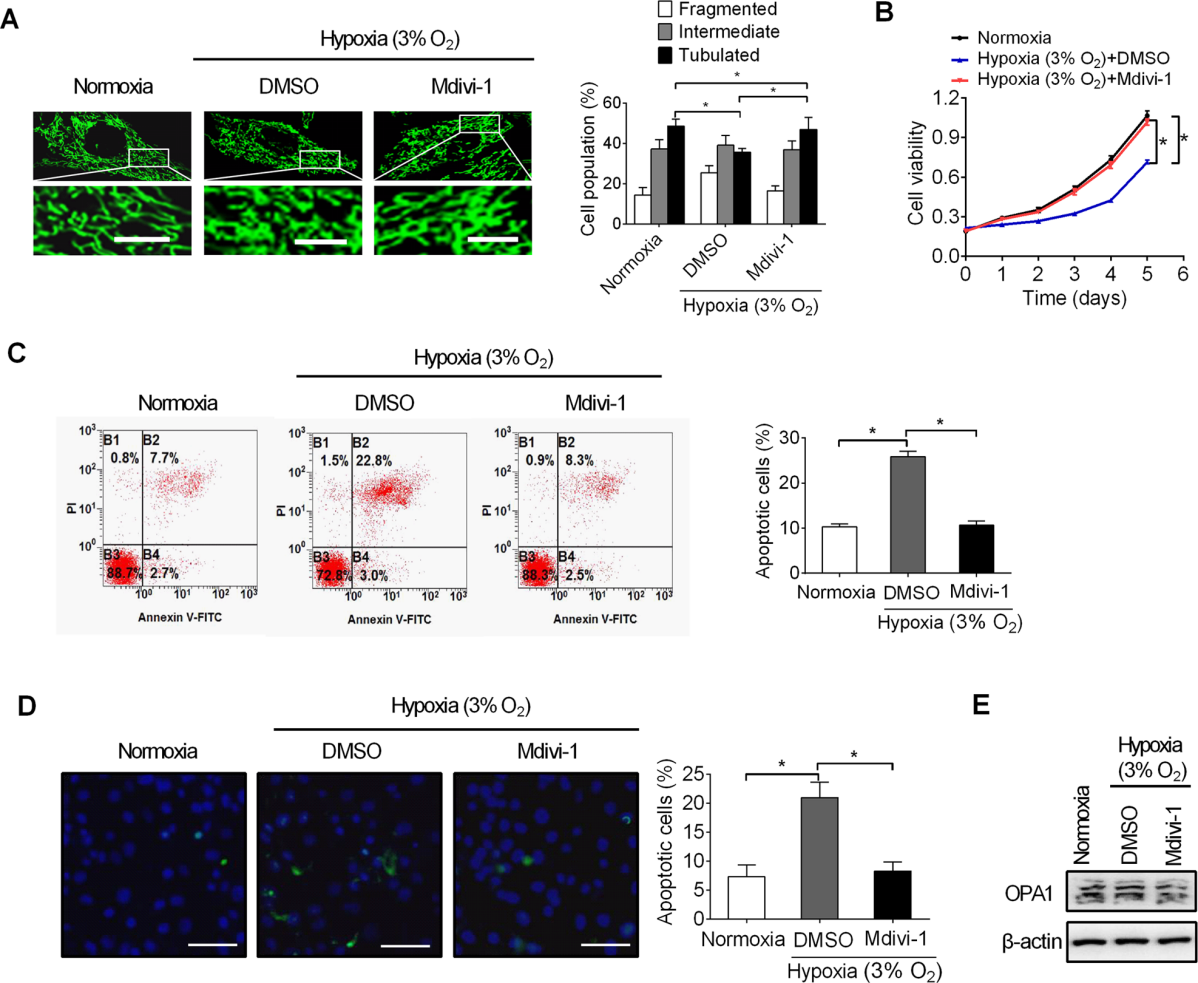
Mdivi-1 exhibits a therapeutic effect on hypoxia-induced cell death of pancreatic beta cells. **A**.Mitochondrial morphologyanalysis by confocal microscopy in pancreatic beta INS-1E cells treated with Mdivi-1 (50 μM) or DMSO for 12 h as indicated.Scale bars, 10 μm.**B.**Cell viability analysis by MTS assay in pancreatic beta INS-1E cells treated with Mdivi-1or DMSO as indicated. **C.**Detection of apoptosis by flow cytometry in pancreatic beta INS-1E cellstreated with Mdivi-1 or DMSO as indicated. **D.** TUNEL staining in pancreatic beta INS-1E cells treated with Mdivi-1 or DMSO as indicated.Scale bars, 50 μm.All experiments were repeated three times.*P< 0.05.

Mdivi-1 is a novel small-molecule inhibitor of mitochondrial fission thatspecifically targets Drp1. To explore the protective role of Mdivi-1 inhypoxia-induced pancreatic betacell death, the effect ofMdivi-1 on mitochondrial fission and cell survivalwere firstly investigated. Ourresults showed thattreatment with Mdivi-1 significantly inhibited mitochondrial fragmentation and clearly increased the viabilityofpancreatic beta INS-1E cells under hypoxia conditions (Fig 5A and 5B).Flow cytometry analysis further demonstrated that Mdivi-1 treatmentsignificantly inhibited apoptosis of pancreatic beta INS-1E cellsunder hypoxia conditions (Fig 5C). Consistently,significantly fewer positive TUNEL staining cells were detected in Mdivi-1 treated pancreatic beta cellsunder hypoxia conditions compared with control by TUNEL assay (Fig 5D). Given the relation between hypoxia and OPA1, we determined the impact of Mdivi-1 treatment on the short and long forms of Opa1 in pancreatic beta INS-1E cells. As shown in Fig 5E, Mdivi-1 treatment did not notably affect the short and long forms of Opa1, suggesting that the protective role of Mdivi-1 in hypoxia-induced pancreatic beta cell death is not mediated by regulation of OPA1. These results collectively indicate that inhibition of mitochondrialfission by the Mdivi-1 is a promising therapeutic strategy for treatment of diabetes.

## Discussion

The increasing evidence supports that hypoxia-mediated cell death is one of the main causes of pancreatic beta cell death, which leads to loss of functional pancreatic beta cell mass and thus type 1 andtype 2 diabetes.However, the functional role of mitochondrial dynamics of fusion and fission, a major cellular stress response, in this processis stillunclear.In the present study, we demonstrated that mitochondrial fragmentation is involved in hypoxia-induced pancreatic beta cell deathpossiblybyfacilitating cristaeremodeling.More importantly, Drp1 knockdown or Mdivi-1 treatment significantly suppressed hypoxia-induced pancreatic beta cell death, which strongly indicating a promising therapeutic strategy for treatment of diabetes.

Recently, cumulative evidence has indicated thatmitochondria dynamics of fusion and fission play a critical role in response tostimuli in the cellular environment. In this study, we observedthat hypoxia treatment significantly increased mitochondrial fragmentation in pancreatic beta cells. Similar results were also obtained in other tissue types from several previous studies.T.H.Sandersonet al. have shown that hypoxia induces excessivefragmentation of mitochondria in isolated primary rat neurons [16]. In addition, Khushbu Jainet al.have alsodemonstratedthat hypoxiainduces alteration of mitochondrial morphology characterized by excessive mitochondrial fragmentation (fission) and decreased mitochondrial fusion in rat brain hippocampus[17].Moreover,WeiZuoet al.indicated that hypoxicconditions can lead to mitochondrial fission and the removal of dysfunctionalmitochondria by autophagyin vascular endothelial cells [18]. Collectively, these results imply the importance of mitochondrial fragmentationin control of pancreatic beta cell death and survival during hypoxia conditions.

Hypoxia-inducible transcription factor (HIF-1α)plays an essential role in cellular adaptation to low oxygen stress.Glenn Marsboom at al. have shown that HIF-1α activation in human pulmonary arterial hypertension (PAH) leads to mitochondrial fission by cyclin B1/CDK1-dependent phosphorylation of DRP1 at Serine-616.Consistently,our present studyalso shows that hypoxia-mediated HIF-1αactivation clearly promotes DRP1 phosphorylation at Ser616 and subsequent mitochondrial fragmentation in pancreatic beta cells.However, wecannot entirely rule out the possibility of other kinases may be involved in this process. Jennifer A.Kashatus et al. have shown that Erk2, another main cellular response to hypoxia, phosphorylatesDRP1 on Serine 616to promote mitochondrial fission in tumor cells[19]. Therefore, whether HIF-1αacts alone or works together with other kinases to promote DRP1 S616 phosphorylation in pancreatic beta cellsduring hypoxia still needs further investigation.In addition, a previous study has shown that hypoxia can impact the 1 cleavage of fusion protein Opa1 in HEK293T and HeLa cells[20]. However, we demonstrated that hypoxia induces mitochondrial fragmentation of pancreatic beta cells not through expressing changes of any key mitochondrial dynamic regulators, but through a more rapid post-translational regulatory mechanism. These contradictions may be explained by the fact that the mitochondrial dynamic regulation under hypoxia conditions is very complicated and diverse regulatory mechanisms exist in different cell types. Cytochrome c is present in the cristae within the mitochondria and itsrelease from the mitochondrial is fatal to the initiation of caspase cascade and apoptosis. In addition, several studies have shown that mitochondrial dynamics of fission and fusion play a critical role in cristae reformation and the pro-apoptotic status of mitochondria.Tatiana Varanitaet al. have reported thatmitochondrial fusionprotein OPA1-dependentcristae remodeling regulates the response of multiple tissues to apoptoticstimuli through inhibitingcytochrome c release from mitochondria[14]. In addition,bothLuca Scorranoet al. and Ryuji Yamaguchiet al. have shownthat mitochondrial fragmentation-mediated mitochondrial cristae remodelingthrough widening of cristae junctions are required for the release of cytochrome c across the outer mitochondrial membrane during apoptosis[21, 22].Moreover, Otera Het al.reported that Drp1-dependent mitochondrial fission regulates cristae remodeling of HeLa cellsduring intrinsic apoptosis [23].Consistently, our studyalso showed that Drp1-mediated mitochondrial fission and cristae remodelingplay an important role in hypoxia-induced pancreatic beta celldeath. Moreover, we found that, under hypoxia conditions, DRP1(S616) phosphorylation-mediated mitochondrial fission was essential for pancreatic beta cell death, which is supported by a previous study in MEF cells demonstrating that mitochondria are fragmentedto facilitate cytochrome c releaseand cell deathduring apoptotic stimuli[24].In contrast, stress conditions such as starvation, UV radiation and oxidation stressoften induce mitochondrial elongation andhyperfusion, which play a protective rolefor cell survival under those stress conditions. These contradictionsmay be explained by the fact that mitochondrial dynamic regulation is very complicated and diverse regulatory mechanisms may exist in different cell types under different environmental stresses.

Mdivi-1 has been well accepted to be a small-molecule inhibitor of mitochondrial fission that specifically targets Drp1. In addition, due to its safety and protective benefits shown in vivo, Mdivi-1 has been shown to represent therapeutics for neurodegenerative diseases and human cancers[25, 26]. However, the ability of mdivi-1 to inhibit Drp1 and impact mitochondrial fission has recently been challenged. Bordt et al did not find any effects of mdivi-1 treatment on mitochondrial morphology in mammalian cells, whereas they found mdivi-1 could inhibit complex I of the electron transport chain and thus exert antioxidant effects. Accordingly, we still cannot rule out the possibility that anti-oxidant effect may also be involved in the protection of Mdivi-1 in hypoxia-induced pancreatic beta cell death, which still needs further investigation.

In summary, our data demonstrate that hypoxia treatment results in a dramatic mitochondrial fragmentation in pancreatic beta cells, which is mediated by HIF-1α activation and subsequent DRP1S616 phosphorylation. Moreover,mitochondrial fragmentation-mediated cristae remodelingplay an important role inhypoxia-inducedpancreatic beta cell death, which could be reversed either by DRP1 knockdown or Mdivi-1treatment, strongly suggestinga promising therapeutic strategyfor diabetes treatment.
